# Early transitions in the evolution of cognition

**DOI:** 10.1007/s40656-025-00709-y

**Published:** 2025-12-03

**Authors:** Arsham Nejad Kourki

**Affiliations:** 1 Francis Crick Institute, 1 Midland Rd, London, NW1 1AT UK; 2 Department of History and Philosophy of Science, Free School Ln, Cambridge, CB2 3RH UK

**Keywords:** Complex multicellularity, Evolution of cognition, Nervous system evolution, Coordination system, Major evolutionary transitions, Structural complexity

## Abstract

This paper examines the early evolution of cognition in animals through the lens of the Transitions in Structural Complexity approach. By focusing on the emergence and transformation of coordination systems, the study identifies three progressive stages: collective, specialised, and integrated coordination. Each stage is characterised by distinct structural innovations—ranging from contractile epithelia and cytoskeletal coupling to the development of neurons, neurosecretory cells, and integrated nervous tissues—and is shaped by the processes of modularisation, subfunctionalisation, and integration. By conceptualising coordination as a transferable biological function, this approach offers an analysis of the evolution of this capacity without presupposing specific mechanisms or structures. Rather than defining cognition in advance, this approach tracks how functions associated with cognition become structurally embodied in multicellular systems through the progressive reorganisation of coordination across levels of biological organisation. Besides offering a general schema for analysing the early stages of cognitive evolution in metazoans, this approach has broader implications for the comparative study of cognition and the structural focus within the major evolutionary transitions framework.

## Introduction and outline

The major evolutionary transitions (MET) framework was initially developed to account for the emergence of new levels of organisation in biological evolution, such as genes, genomes, cells, and multicellular organisms (Maynard Smith & Szathmáry, 1995; Szathmáry, 2015). Over time, this framework has been extended into new domains, including the evolution of cognition and sociocultural organisation. This paper focuses on the former. The aim is to demonstrate the utility of the Transitions in Structural Complexity (TSC) approach—developed in recent theoretical work (Madhani & Nejad Kourki, 2024; Nejad Kourki, 2022)—for understanding early steps in the evolution of cognition in metazoans. In doing so, the paper also advocates for a more open and integrative approach to the MET framework, one that can accommodate the evolution of biological units at all levels of organisation, not solely the emergence of new evolutionary individuals.

Discussions of the evolution of cognition are frequently tied to the evolution of the nervous system. Even when the concept of ‘minimal cognition' is invoked—referring loosely to cognition without a centralised nervous system or in non-neural organisms—attention tends to remain focused on systems that already resemble those found in animals (Lyon et al., 2021). Nevertheless, the evolution of the nervous system has long been recognised as a plausible candidate for a major transition. Jablonka and Lamb (Jablonka & Lamb, 2006) were among the first to argue explicitly for its inclusion, and Szathmáry has since suggested that he and Maynard Smith had considered it for a revised list of METs prior to the latter's passing (Calcott & Sterelny, 2011). More recently, several authors have developed theoretical approaches to studying the evolution of cognition itself in terms of a sequence of local METs. Of particular interest here is the Major Transitions in the Evolution of Cognition (MTEC) approach developed by Barron, Halina, and Klein, which identifies five such transitions in the metazoan lineage: the emergence of (1) networks of neurons, (2) centralised nervous systems, (3) recurrence, (4) lamination, and (5) reflection (Barron et al., 2023). While this schema captures key organisational changes, its primary focus is on structural changes that allow shifts in information control flow processing, and the first two stages correspond to identifiable episodes in early metazoan evolution.

The argument developed here is that the TSC approach not only complements but also deepens the MTEC schema. First, it situates the early evolution of cognition within the more general domain of structural reorganisation—specifically, the emergence of new intermediate units of organisation. In the present context, these structural reorganisations are those that allow changes in information architecture. Second, it identifies a previously overlooked 'transition zero': the evolution of collective coordination, which precedes the appearance of neural networks but sets the stage for them. Third, it aligns the first two MTEC transitions with what can be termed specialised and integrated coordination, respectively, providing a unifying vocabulary for the functional and structural shifts involved. In this schema, collective coordination refers to a state in which unspecialised cells coordinate behaviour through broadly distributed means, typically without specialised coordinators; specialised coordination emerges when certain cells (e.g., neurons or neurosecretory cells) evolve to take on dedicated coordination roles; and integrated coordination arises when those cells form higher-level units, such as tissues or organs, to enhance efficiency and scale. This framework builds upon the processes of modularisation, subfunctionalisation, and integration, as outlined in the TSC approach. I begin the paper by discussing the relationship between coordination and cognition (Sect. 2.1) followed by a refresher on the TSC approach (Sect. 2.2) and then linking the two together (Sect. 2.3). I will then explain each of these stages (collective, specialised, and integrated coordination) in some detail within the context of early metazoan evolution (Sects. 3.1–3.3). I then discuss what comes beyond the three stages (Sect. 3.4) before concluding the paper (Sect. 4).

## Cognition, coordination, and evolutionary transitions

### Cognition and coordination

Cognition, in the traditional sense, refers to a set of internal processes—typically assumed to involve the formation and manipulation of representations—that enable an organism to perceive, interpret, and respond to features of its environment. These processes are commonly taken to include memory, learning, decision-making, and goal-directed behaviour, and are often assumed to depend on the presence of a nervous system (Adams, 2018; Shettleworth, 2009; Van Duijn et al., 2006).

The notion of *minimal cognition* has emerged as a response to the tendency to restrict cognition to a narrow subset of animals with complex nervous systems (Van Duijn et al., 2006; Keijzer, 2015). This notion sits neatly within the *basal cognition* research programme, which invites us to consider a broader array of biological systems—especially non-neuronal organisms such as slime moulds, plants, and bacteria—as potentially exhibiting cognitive capacities (Hanson, 2023; Lyon & Cheng, 2023; Reid, 2023; Seifert et al., 2024; Sims, 2024). The proponents of the programme have thus often put such broad notions of cognition in terms of ‘minimal’ or ‘basal’ cognition. This move not only counters anthropocentric and neurocentric biases but also reflects a growing recognition that many of the mechanisms associated with cognition in animals—whether homologous or not—may have evolutionary antecedents or functional analogues in simpler organisms (Fábregas-Tejeda & Sims, 2025).

At the same time, however, this expansion raises an important worry. As Fábregas-Tejeda and Sims caution, the further we stretch the concept of cognition, the greater the risk of obscuring the boundary between cognitive and more general adaptive or physiological processes. If cognition is equated with any form of responsiveness, regulation, or information processing, it becomes unclear what distinguishes it from the rest of life’s functions. This risk becomes especially acute when capacities such as memory or learning are defined so broadly as to encompass everything from synaptic plasticity to gene regulation. The result is a conceptual sprawl that undermines explanatory clarity and weakens the force of comparative claims.

A helpful way of framing this issue—introduced by Fábregas-Tejeda and Sims—is in terms of *oligoextensionalist* and *panextensionalist* positions regarding the phylogenetic scope of cognition. The former limits cognition to a small set of taxa, typically animals with nervous systems; the latter, in its extreme form, extends it across all living systems. Each extreme brings characteristic risks: oligoextensionalism may blind us to deep evolutionary continuities and to important forms of coordination in non-neural organisms; panextensionalism, by contrast, risks flattening those distinctions entirely, leaving us with a concept of cognition so diffuse as to be explanatorily inert. It is important to point out that Fábregas-Tejeda and Sims argue not merely for a compromise between these poles but for a middle ground rooted in evolutionary reasoning. Rather than resolving the scope of cognition by definitional stipulation, they propose treating it as an open empirical question—one to be approached through phylogenetic, mechanistic, and comparative investigation. On this view, the task is not simply to draw a boundary, but to locate the evolutionary joints in the nature of cognition: the points at which cognitive capacities diverge, consolidate, or reconfigure across lineages and levels of organisation. Some enactive and autopoietic accounts intentionally collapse the distinction between cognition and metabolism, treating them as continuous aspects of self-maintenance (Di Paolo, 2018; Keijzer, 2021; Thompson, 2007). While such approaches have their merits, my aim here is different: to preserve coordination as a narrower, functionally tractable category. By resisting a full merger with metabolism or development, I follow the oligoextensionalist strategy of keeping the scope of cognition selective rather than maximally broad. This allows the TSC approach to distinguish coordination from other adaptive processes while still leaving open multiple theoretical interpretations.

This is precisely why I have preferred the term *coordination* throughout. I use coordination to mean the capacity of biological systems to regulate their behaviour as a whole and that of their constituent parts in light of internal and environmental signals. At its core, coordination involves the establishment of control-flow architectures: semi-independent components whose activities are modulated by specialised mechanisms so that their collective behaviour supports the viability of the whole. This notion applies at multiple levels of organisation, from local tissue-level regulation to whole-organism integration, and it differs from mere stimulus–response coupling in that the signal pathways form part of an internal regulatory architecture rather than being imposed unilaterally by external conditions. The emphasis on control-flow architectures resonates with the MTEC account of cognitive evolution developed by Barron et al. (2023), which tracks major transitions in the computational organisation of nervous systems. My focus here, however, is broader: I extend this control-flow framing to earlier multicellular contexts, identifying forms of coordination that predate nervous systems but set the stage for their emergence.

This is a functional description, in the sense common to biology when we attribute functions to organs or tissues. One might worry, however, that to describe coordination functionally is already to commit to a computational ontology of cognition, since in cognitive science functional characterisations are often taken to imply information-processing. Piccinini and Scarantino (2011) note that this assumption is widespread but contentious: functional and computational descriptions are not coextensive, even though in many areas of cognitive science the two are routinely conflated (Piccinini, 2007). My account is consistent with a computational reading, since coordination can indeed be modelled as an information-processing architecture (Barron et al., 2023; Barsalou et al., 2007; Kirsh, 2008), but it does not depend on such a reading. The same functional description is equally compatible with enactive and autopoietic interpretations, where coordination is the organisation of sensorimotor and physiological couplings that sustain agency (De Jaegher & Di Paolo, 2007; Froese & Di Paolo, 2011). On both readings, what matters is the same architectural question: who regulates what, and by which channels? I also emphasise that Neutrality here does not mean theory-free vagueness. To characterise coordination functionally is already to adopt a certain mode of description—one that highlights architectural roles and interactions. My claim is that this level of description is sufficiently general to be consistent with multiple ontological commitments. Coordination thus sits at an intermediate level: above specific mechanisms such as neurons or hormones, but below the more theory-laden concept of cognition itself.

This functional-architectural framing provides a neutral entry point into the problem space, by avoiding some of the baggage often attached to cognition (e.g. commitments to representation, consciousness, or neural chauvinism). It allows us to track how the capacity for adaptive regulation shifts and scales—particularly in the transition to complex multicellularity—without requiring that every instance be immediately classified as cognitive. In this sense, higher-level coordination can serve as a functional proxy for cognition, enabling analysis of how coordinative architectures evolved in early animals while leaving open the precise cognitive ontology one prefers (Godfrey-Smith, 2016; Sims, 2023; Sims & Yilmaz, 2023).

On this basis, I propose that what I call a *synthetic approach* to cognition would be the most fruitful: one that recognises the plurality of ways the concept is currently used but seeks to organise and integrate these uses through an evolutionary and organisational lens. Different senses of cognition may vary in their *intension*—the mechanisms or traits they invoke—and in their *extension*—the kinds of organisms or systems to which they apply. These senses need not be forced into a single definition, but they can be compared, related, and situated within a broader explanatory framework. This allows us to move beyond catch-all categories like ‘minimal cognition’ and instead map different cognitive phenomena to different forms and degrees of coordination, thereby offering a richer and more tractable taxonomy. One of the key advantages of this approach is that it supports more precise explanatory and predictive claims. For instance, by restricting the scope of cognition to certain kinds of relatively rapid, reversible coordination processes—typically mediated by chemical or electrical signalling—we can draw useful distinctions between such processes and slower, developmentally embedded forms of adaptiveness. This clarification also helps to preserve the link to phenomena such as learning and memory, which typically rely on the interaction of rapid coordinative processes with slower, developmentally embedded forms of plasticity. This does not mean excluding the latter from biological explanation but rather clarifying the kinds of mechanisms we are referring to when we speak of cognition.^1^ In this way, the synthetic approach preserves conceptual openness while offering tools for discriminating between overlapping but distinct phenomena.

### Transitions in structural complexity

This brings us directly to the *Transitions in Structural Complexity* (TSC) approach, which provides a theoretical basis for understanding how novel forms of coordination emerge—not only in terms of what changes, but in terms of how and why those changes take place. In the sections that follow, I focus in particular on the evolution of coordination in the context of complex multicellularity in animals. This includes not only the specialisation of particular cell types for coordination but also the progressive transfer of behavioural control from individual cells to the organism as a whole. Understanding this process provides a basis for explaining the emergence of more complex cognitive capacities—grounded in the logic of evolutionary transitions and complementing the lens of the MTEC framework more focused on information control flow architectures.

The Transitions in Structural Complexity (TSC) approach (Madhani & Nejad Kourki, 2024; Nejad Kourki, 2022) addresses how biological systems acquire new forms of internal structure and functional integration. Unlike the Evolutionary Transitions in Individuality (ETI) approach—which focuses on the emergence of new units of selection through reproductive reorganisation—the TSC approach targets the emergence of units of organisation: tissues, organs, and intermediate systems that enable cohesion and functionality at higher levels, irrespective of whether they act as units of selection.

At the heart of this approach are four abstract evolutionary processes: modularisation, subfunctionalisation, integration, and deletion. These processes are not treated as a fixed sequence, nor as direct developmental mechanisms. Instead, they provide a conceptual vocabulary for tracking how biological systems become more differentiated and more structurally coherent across levels of organisation. Modularisation generates repeated units; subfunctionalisation diversifies those units into specialised forms; integration assembles parts into new wholes; and deletion removes them.^2^ These processes often operate in parallel and across different scales, contributing to the emergence of what we recognise as new levels of organisation. Levels of organisation, in this context, are not understood as being occupied by units of selection but by *units of* organisation characterised by their primarily structural but often also function individuation, which aligns with accounts that treat levels as sites of emergent organisation rather than selection (Brooks et al., 2021). The TSC approach builds on this perspective by offering tools to track the transformations through which these units and levels emerge, including cases where intermediate units themselves become substrates for further structural innovation.

A key feature of the TSC approach is the claim that what explains the emergence of new units of organisation that are not units of selection (e.g. tissues and organs)—ultimately in adaptive terms—is the transfer or emergence of functions from one level of organisation to another. Simply put, core biological functions such as digestion, transport, coordination, etc. are transferred or emerge de novo at an emerging higher level of organisation such as that of the multicellular organism, which tends to require the evolution of intermediate (i.e. intermediate between the lower and higher levels of selection in the levels hierarchy) units such as tissues and organs.

### Coordination, structural complexity, and cognition

This paper focuses on coordination as a case of function transfer. As defined above it is not intrinsically tied to any one structure or level but can be implemented through a range of mechanisms, from local feedback loops to large-scale control systems. This becomes particularly significant in the context of complex multicellularity, where increased interdependence among parts places greater demands on systemic regulation. Cells in such organisms must act in concert to maintain functional integrity, and the emergence of coordination systems becomes a crucial evolutionary solution. The TSC approach proves especially useful here: it enables us to track how coordination functions are progressively reorganised and structurally embedded in increasingly complex architectures, particularly during the early evolution of nervous and endocrine systems. The core processes of modularisation, subfunctionalisation, integration, and deletion offer a conceptual toolkit for understanding how distributed mechanisms of regulation first emerge and then give rise to more centralised and functionally differentiated systems—thereby setting the stage for the emergence of cognitive capacities in the stricter sense, grounded in more basic mechanisms of system-level control.

Early metazoan evolution is particularly well suited for examining this purpose. As I show in the following sections, the transition from collective to specialised to integrated coordination in early metazoan evolution maps neatly onto an increase in the structural complexity and organisational scale of coordination systems. The latter two stages—developed here as an extension of the TSC approach—also align with the first two stages of the MTEC schema, allowing for a direct theoretical connection between the two perspectives. By adding what I term a ‘transition zero’ to the MTEC framework (emergence of the collective stage), the TSC approach can help us better understand how cognitive capacities evolve from simpler forms of coordination. In what follows, I examine the suggested stages in the evolution of coordination—collective, specialised, and integrated—in detail. The analysis is restricted to coordination systems in multicellular animals, and in particular to neuroendocrine systems. This is partly because animals are the paradigmatic cognitive organisms, and the main point of interest here is to understand the earliest steps in the evolution of cognition, even though I acknowledge that similar steps may have been taken in members of other clades on which I lack appropriate expertise.

The relationship between the two frameworks can be put more precisely by noting that the stages of coordination I describe—collective, specialised, and integrated—are functional-architectural states: ways in which control-flow is organised across a multicellular system. By contrast, the four processes identified in the TSC framework—modularisation, subfunctionalisation, integration, and deletion—are transformational processes operating on structural units such as cells, tissues, or organs. The mapping between the two is a way of showing how structural transformations at one level (e.g. the modularisation of cell clusters, or the subfunctionalisation of neural precursors) give rise to new forms of coordination at another. This makes it possible to align the structural changes described by the TSC approach with the functional transitions identified in the MTEC, and to see them as two complementary descriptions of the same evolutionary phenomena.

## Collective, specialised, and integrated coordination

In this section, I detail a sequence of evolutionary stages—collective, specialised, and integrated coordination—that describe how coordination systems emerged in early metazoan evolution. The central idea is that increasingly complex collectives demand more effective forms of coordination, achieved through the gradual transfer of coordinative capacity to higher levels of organisation. This extends the TSC approach by linking structural transformation to changes in how coordination is implemented. As discussed above, I use the term coordination to mean cases where semi-independent units (cells, tissues, or organs) adjust their activity through physiological signalling so that their collective behaviour maintains or enhances the viability of the whole. And I use the term ‘coordinators’ to refer to structural and functional units that are specialised for carrying out coordination tasks. Neurons are the most familiar example: they are units whose morphology and physiology are dedicated to transmitting and integrating signals, thereby enabling coordination either locally (within tissues) or globally (across the whole body). More generally, coordinators are constrained by their embedding in a larger system: their function depends on and co-evolves with the other units they regulate and are regulated by. In this sense, the coordinators are the signalling mechanisms themselves—initially chemical, later also electrical and hormonal—and what is being coordinated are the activities of those semi-independent units. This framing avoids the definitional difficulties of ‘cognition’ while being more specific than broader notions such as regulation or information processing. This makes it suitable for tracking how coordination becomes embedded in biological structure over evolutionary time.

The three stages are cumulative: specialised coordination presupposes collective coordination, and integrated coordination presupposes specialised parts that can be assembled into larger systems. At each stage, what is coordinated are the activities of increasingly complex units, and who coordinates shifts from broadly distributed signalling (collective), to dedicated cell types (specialised), to higher-level tissues and organs (integrated). They are distinct from the core TSC processes—which recur in many contexts—but shaped by them: collective coordination depends on basic integration and subfunctionalisation; specialised coordination on further subfunctionalisation and modularisation (e.g. spatially organised nerve nets) and integrated coordination on the assembly of specialised parts into organs. The analysis that follows focuses on neuroendocrine systems in animals, for three reasons: this is the focus of the MTEC framework; it corresponds to a canonical organisational transition (unicellular to multicellular); and it offers a concrete setting in which to address the evolution of cognition.

The following sections trace how coordination functions are progressively reorganised through structural changes in early metazoans. Each stage represents not only the presence of new coordinative mechanisms but also a reorganisation of how pre-existing functions are distributed and scaled.

### Collective coordination

#### What is collective coordination?

I define **collective coordination** as the activity whereby constituent units of a system coordinate their behaviours through mechanisms of overall consensus—that is, through broadly distributed signalling and response dynamics (such as quorum sensing, chemical gradients, or ciliary beating) in which each unit adjusts its behaviour in light of the others—without relying on any subset of components specialised for that function. In these systems, coordination emerges from transient, distributed interactions among equivalent units—each contributing more or less equally to the collective behaviour. This mode of coordination tends to be relatively slow and spatially restricted, typically relying on local mechanisms such as paracrine signalling or mechanical contact between adjacent cells.

Collective coordination is especially relevant to the earliest stages of multicellular evolution. It provides a way for organisms to regulate whole-body behaviour using existing molecular processes, without requiring the emergence of neurons, endocrine cells, or other specialised parts. Despite this significance, it is sometimes overlooked in accounts of nervous system evolution, which tend to begin with already-specialised components [see Keijzer et al. (2013) for a similar argument]. I suggest instead that collective coordination constitutes a distinct evolutionary transition—what I call *transition zero* in resonation with the MTEC approach—which sets the groundwork for later stages by relocating regulatory capacity to the level of the emerging multicellular whole. It is worth noting here that although development also includes a set of processes involving intercellular coordination, my focus here is on physiological coordination, which I define as relatively rapid and reversible processes that respond to transient ecological conditions, in contrast with developmental coordination, typically understood as involving slower and more permanent changes such as shifts in gene expression that underlie cell states, or tissue structure. Distinguishing these two forms of coordination is important not only conceptually but also in tracing how physiological regulation, in particular, scales with increasing complexity.

The suggested emphasis on collective coordination is consistent with several recent proposals that shift emphasis away from traditional input–output models. Keijzer’s Skin-Brain Thesis (SBT) suggests that nervous systems originally evolved to coordinate contractile surfaces in multicellular organisms, not to process sensory input for motor outputs (Keijzer, 2015; Keijzer et al., 2013). Jékely’s Chemical Brain Hypothesis (CBH) similarly proposes that body-wide, chemically mediated signalling networks—especially peptidergic systems—preceded synaptic organisation and tiled the body surface to coordinate cellular activity (Jékely, 2021; Jékely et al., 2021). Both views converge on the idea that system-wide coordination was a primitive function of early nervous systems, and that its precursors were already at work in simpler multicellular organisms. The present suggestion supplements by placing the emphasis on pre-neural system-wide coordination.

### Collective coordination in metazoan evolution

We see prime examples of multicellular organisms relying principally, if not exclusively, on collective coordination in the closest multicellular relatives of metazoans—colonial choanoflagellates—as well as two early-diverging animal phyla: the sponges (Porifera) and placozoans (Placozoa). In all three cases, the most prominent short-term changes mediated by physiological adaptive processes at the collective level are changes to body shape, and in all three cases these changes are directly linked to feeding behaviour (Brunet et al., 2019; Elliott & Leys, 2007; Kirkegaard & Goldstein, 2016; Kirkegaard et al., 2016; Leys & Meech, 2006; Ueda et al., 1999; Varoqueaux et al., 2018).

Choanoflagellates first. Members of the clade Choanoflagellata, our closest non-metazoan relatives, comprise both unicellular and multicellular forms, and considerable evidence supports the hypothesis that the earliest metazoans were very similar to colonial choanoflagellates (Brunet & King, 2017). Though the colonial choanoflagellates themselves only form colonies smaller than any modern sponge, they do exhibit collective coordination in the way they change their individual cell shapes which results in emergent colony-level shape change, and thereby affects the flow of water around the colony (Brunet et al., 2019; Kirkegaard & Goldstein, 2016; Kirkegaard et al., 2016). Influencing and controlling the flow of water is of utmost importance to colonial choanoflagellates because they are *suspension feeders*: they filter flowing water and extract bacteria, which their individual cells then ingest. Controlling the flow of water around the colony has a direct effect on the efficiency of this mode of feeding, which additionally relies heavily on ciliary beating for modulating the flow of water.^3^ This provides a simple but telling example of collective coordination: individual cells adjust their ciliary activity in response to local flow conditions, and together these decentralised adjustments determine the colony’s overall feeding performance. In this way, feeding efficiency depends not only on the action of individual cells but on their capacity to act in concert, a precursor to more elaborate coordinative mechanisms in later metazoans. Curiously, we see here the first signs of association between mechanisms underlying collective coordination and mechanisms of shape change underlying muscular action, since choanoflagellates utilise the same elements of the cytoskeleton for shape change—i.e. actin microfilaments and myosin (Brunet et al., 2019; Pasha et al., 2016; Sebe-Pedros et al., 2014)—as muscle cells rely on across the eumetazoans.

Now onto sponges. Members of the clade Porifera (Latin, ‘pore-bearers’) are likely the sister-group to all other metazoans^4^ and their general morphology (though diverse) can basically be characterised as a macroscopic, bilayered, and often highly compartmentalised choanoflagellate colony with an internalised cavity (or interconnected cavities) that is highly adapted for efficient suspension feeding. Despite their macroscopic size, sponges typically only have a handful of cell types and whether these cells constitute tissues is itself a matter of debate (Adams et al., 2010; Leys & Hill, 2012; Leys et al., 2009); in other words, sponges make do with very little specialisation of parts, which marks the fact that their constituent cells are not strongly interdependent.^5^ Nevertheless, they do exhibit a remarkable capacity for collective coordination that is plausibly a direct continuation of the corresponding capacity in choanoflagellate colonies: body-wide contraction without specialised contractile cells. While sponges frequently change their body shape to modulate the flow of water in their internal cavities via their collective contraction mechanisms, this behaviour is more most clearly seen in ‘sneezing’ behaviour (Kornder et al., 2022).^6^ Thus, sponges exhibit a more accentuated version of the kind of collective coordination mechanism found in choanoflagellate colonies, which similarly relies on relatively restricted processes of paracrine signalling between cells, which ends up being quite slow for macroscopic organisms like sponges (relative to the coordination mechanisms of animals with nervous systems, as we shall see below).

**Fig. 1:**
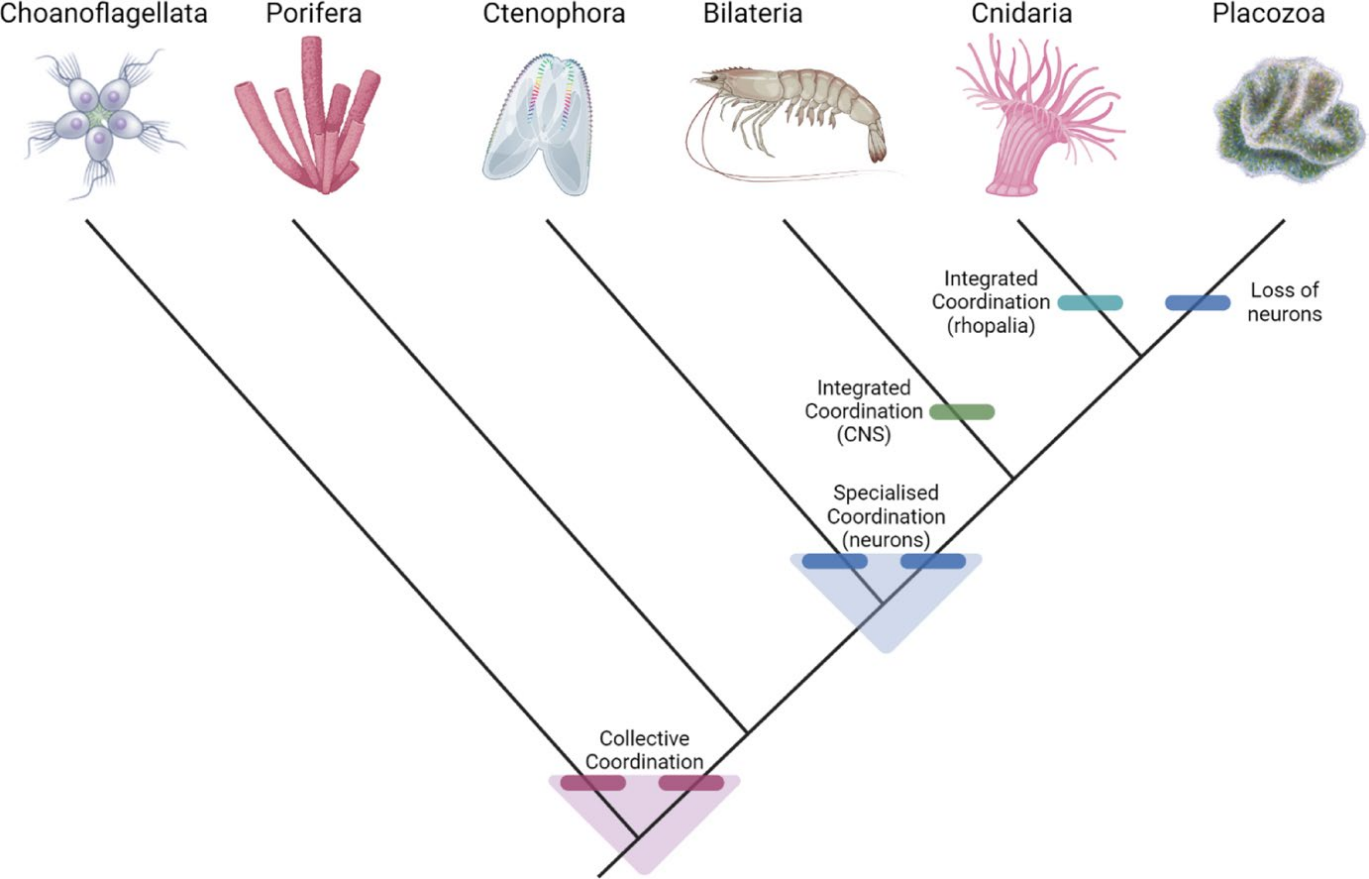
The evolution of collective, specialised, and integrated coordination in early metazoan evolution. Collective coordination is present in both metazoans as well as colonial choanoflagellates and may therefore have evolved either independently in the two lineages or was present, in some form, in their last common ancestor. Either way, the components needed to make it happen was present in the last common ancestor and facilitated its evolution, hence the transparent area in pink. Similarly, neurons may or may not have been present in the last common ancestor of ctenophores and other eumetazoans or they may have evolved independently; again, either way, the components needed for their evolution were largely present in their last common ancestor, hence the transparent area in blue. Integrated coordination has evolved independently in the bilaterians and cnidarians. Placozoans have likely secondarily lost their neurons, in case they are indeed the sister group to cnidarians. Phylogeny after (Laumer et al., 2018). Figure made using BioRender

And now placozoans. The clade Placozoa consists of the three described genera *Trichoplax*, *Hoilungia*, and *Polyplacotoma* (Eitel et al., 2013, 2018; Osigus et al., 2019; Schierwater et al., 2021). These are microscopic, bilayered, dorsoventrally flattened ciliated animals with uncertain phylogenetic affinities.^7^ Regardless of their phylogenetic placement and its implications for the polarity of their key characters, they also exemplify the same kind of collective coordination that is found in sponges and colonial choanoflagellates: change of body shape to modulate feeding (Jackson & Buss, 2009; Ueda et al., 1999; Varoqueaux et al., 2018). But there are two main differences between placozoans and sponges in this regard. One is that, strictly speaking, placozoans are not suspension feeders. Instead, they feed on microorganisms living on surfaces (such as the seafloor or an aquarium wall). They do this by moving around on these surfaces using their ciliated lower surface, finding where microorganisms are more concentrated, and creating a temporary ‘digestive sac’ underneath them via changing their body shape, secreting enzymes into the digestive sac and absorbing the digested material (Sperling & Vinther, 2010). The second main difference lies in *how* they achieve this change in body shape: to achieve this, they primarily use their upper epithelium, which consists of contractile cells that communicate with each other using chemical paracrine signals (Varoqueaux et al., 2018). Thus, there is a small degree of specialisation in coordinative functions in placozoans, but, as far as we know, no cells that are fully specialised for this function since the upper epithelium also plays other roles for placozoans (see Fig. 1). This case exemplifies collective coordination, since the contractile epithelium contributes to coordination without being fully specialised for that role.

As we will now see, the emergence of specialised coordinators such as neurons and neurosecretory cells represents a reorganisation of coordination functions: control shifts from broadly distributed signalling among unspecialised cells to the delegation of coordination to dedicated cell types.

### Specialised coordination

#### What is specialised coordination?

As we have seen, Collective coordination in biological systems, such as those found in animal groups, involves intricate interactions that rely heavily on mechanisms of intercellular communication, such as paracrine signalling, quorum sensing, or the propagation of electrical or chemical gradients. These processes can be described functionally as mechanisms of information transfer, but here I use the term in a neutral, biological sense, without implying any particular ontological commitments (e.g. computationalism). These signalling mechanisms, however, face intrinsic limitations, particularly at larger size scales. For instance, in the context of paracrine signalling, signals are often slow to propagate and may become diluted or lose specificity over distance, reducing the efficiency of response in larger groups or across extended spaces. This limitation impacts the ability of groups to synchronise behaviours and make unified decisions effectively. One key aspect is the challenge of maintaining cohesive group behaviours when relying on local and often slow communication mechanisms, which can lead to delays in response to environmental changes or threats (Ouellette & Gordon, 2021). Moreover, as group size increases, the complexity and cost of maintaining effective communication can lead to decreased coordination efficiency, which might limit the size of stable groups or the complexity of tasks they can handle efficiently (Couzin, 2014).

As early macroscopic animals faced increasingly complex adaptive challenges that needed increasingly rapid responses (especially the need to catch prey and avoid being preyed on), and as their cells became more interdependent and specialised for a variety of different functions, some of their cells accordingly specialised for coordination functions both locally (within a part of the organism) and, especially, globally (for the whole organism). This comprises specialised coordination, which is exemplified by the evolution of both neurons and later endocrine cells.^8^ Specialised coordination differs from collective coordination in that certain cells evolve to take on dedicated coordinating functions, rather than performing coordination as one task among others. The evolution of specialised coordination effectively bypasses the need for coordination to have to go through most (or a very large proportion of) cells in the multicellular organism (which is a relatively slow process), making coordination more efficient in the same general way that any sort of specialisation increases functional efficiency at the higher level, and ultimately reinforces the increase in interdependence and the corresponding decrease in the autonomy of individual cells in multicellular organisms. Once again, my focus here is on metazoan evolution, and I will therefore forego other possible cases of specialised coordination at the multicellular level, as well as parallel examples in transitions to other levels of individuality (e.g. eukaryotic or superorganismal).

### Specialised coordination in metazoan evolution

Though there is some uncertainty about whether neurons were present in the last common metazoan ancestor, it is abundantly clear that while sponges and placozoans lack this cell type (Moroz & Romanova, 2022), cnidarians and bilaterians (the unequivocal eumetazoans) have neurons that are almost certainly homologous (Kelava et al., 2015), and that ctenophores also have this cell type though its homology with the neurons of unequivocal eumetazoans is unclear (Dunn et al., 2015). Neurons play a crucial role in giving rise to a system for specialised, rapid, and highly efficient information processing for the means of coordination across the whole multicellular organism in the eumetazoans—despite energetic, metabolic, developmental, and adaptive costs associated with their emergence (Keijzer, 2015). Leading research suggests that the emergence of neurons as specialised coordinators is tightly associated with the evolution of specialised sensory and locomotory cells and tissues. Arendt and collaborators have proposed that the first nerve nets appeared for facilitating tissue coordination underlying contractile or cilia-driven movements, emphasising the influence of early animal body plans and feeding behaviours, such as mucociliary mechanisms and gastric pouches, on the evolution of the nervous system (Arendt et al. 2015; Arendt, 2021). Furthermore, Jékely has suggested that early nervous systems evolved from chemically organized networks before synapses developed, with peptidergic signalling linking cells into networks and enabling complex neuronal functions. Another hypothesis by Jékely suggests that neural circuits evolved to enhance sensory-motor transformations—that is, the conversion of sensory inputs (such as light, chemical or mechanical cues) into coordinated motor outputs (such as changes in ciliary beating patterns)—in early ciliated organisms, thereby enabling more complex behaviours (Jékely, 2011, 2021). Together, these cell types and their corresponding behavioural traits comprise an effective—and, crucially, adaptive—input-process-output system^9^ found virtually in all eumetazoans with varying degrees of complexity, ranging from simple circuits that mediate classic examples of habituation to the sophisticated centralised nervous systems found in certain bilaterian clades (see below: Sect. 2.3).

Though the majority of nervous systems nowadays are integrated to various degrees due to the remarkable evolutionary success of the bilaterians (whose success is in part explained by their complex nervous systems), specialised nervous systems lacking considerable integration are found in ctenophores, cnidarians, and some bilaterians (e.g. xenacoelomorphs and platyhelminths). The ctenophore nervous system is characterised by a subepithelial nerve plexus, a diffuse network of neurons located just below the epidermis in the form of by a continuous plasma membrane forming a syncytium (Burkhardt et al., 2023); as well as the mesogleal nerve net which includes features such as anastomoses between neurites of the same cell through a continuous membrane, a unique characteristic that has been suggested to facilitate complex neural integration without the conventional synaptic structures found in other animals (Norekian & Moroz, 2020). The homology relation between ctenophore subepithelial neurons with the neuroepithelial cells of cnidarians and neurons of bilaterians is unclear in part due to this unique structure as well as the utilisation of a distinct set of synaptic proteins in addition to the uncertain phylogenetic placement of ctenophores and their high rate of evolution as a whole (Moroz, 2015; Whelan et al., 2017; Zhao et al., 2019). As their name suggests, the neuroepithelial cells of cnidarians are embedded within their epithelial tissues,^10^ where they form reticulated networks not at the level of individual cells like ctenophore neurons do, but at the level of the tissue. There is generally little integration, though they do form ganglia, a circumoral ring, and on some occasions locally integrated sensory structures known as rhopalia (Sprecher, 2022). Cnidarian neurons communicate using a combination of neuropeptides (Hayakawa et al., 2022) electrical synapses (gap junctions) and chemical synapses (using neurotransmitters), and while gap junctions are utilised elsewhere (e.g. placozoan contractile epithelium, vertebrate cardiac muscle) chemical synapses are generally restricted to signal transduction between neurons on one end and other neurons, sensory cells, or effector cells (muscular or endocrine) on the other end. This suggests that even though synaptic components were already present in the last common metazoan ancestor (evidenced by their presence in sponges) their unique integration into chemical synapses evolved either with the emergence of neurons themselves or in the hypothesised neuronal precursor cell type (neuromuscular/neurosensory).

### The endocrine system

It is now important to emphasise that neurons *are not the only cell type used for specialised coordination* of metazoan behaviour (particularly motility): the other set of cell types specialised for this function is, arguably, that of endocrine cells. The nervous and endocrine systems have the same general function of coordination at the multicellular level, though the endocrine system bears some distinct features. The first of these features is that the endocrine system operates at a much broader range of timescales. Disregarding the vast array of effects that the endocrine system has on development and restricting ourselves to physiological changes, this is at times comparable to and closely associated with the actions of the nervous system (minute scale), and at other times has longer-term effects (hour to day scale). A prime example of the former is the release of epinephrine and norepinephrine by both the nervous system and the endocrine system in vertebrates in giving rise to the fight or flight response: while neurons can activate this response rapidly (within milliseconds to seconds), the slower release of these hormones by the endocrine system last longer (on a timescale of seconds to minutes and lasting up to hours), which explains why being frightened by a light stimulus does not make one tremble for minutes (or even hours) in the same way that getting in a seriously dangerous situation often does (Romero, 2010). Another interesting feature of endocrine cells is that it can only operate in case the organism has an effective means of rapidly (relatively speaking) distributing the hormones that it produces. This is typically achieved by the circulatory system possessed by more active and/or larger animals.

Thus, the emergence of neurons and (likely later) endocrine cells characterises not only the transition to specialised coordination but also the first transition in the MTEC schema: the emergence of network-shaped information flow architecture. It is because of this correspondence that I consider the evolution of collective coordination as a ‘transition zero’ for the MTEC schema: there can be no specialized network without there first being a collective effort for coordination. Specialisation evolves to make coordination more efficient. Moreover, specialisation here has been defined such that it automatically translates to a difference in the flow of information: whereas in collective coordination information flows more diffusely across most components (e.g. cells) of the system (the whole organism) or a large part thereof (e.g. contractile epithelium), in specialised coordination networks such as nervous or endocrine system are set up such that the flow of information is more restricted and more regulated (Table 1).

**Table 1:** Stages of coordination and examples of TSC processes involved at each stage. Each process contributes differently across stages to the emergence of increasingly complex coordination systems in metazoan evolution

Stage/process	Modularisation	Subfunctionalisation	Integration
Collective		Contractile epithelium in placozoans	Cytoskeletal coupling in choanoflagellates and sponges
Specialised	Regular spacing of nerve nets (Jékely: “tiling”)	Evolution of neurons and neurosecretory cells	Evolution of neurosensory organs without centralised nervous systems (e.g. rhopalia)
Integrated	Ganglia and cords with repeating structures	Diversification of integrated neuroendocrine structures	Evolution of centralised nervous systems (nerve cords)

We will now look at integrated coordination, which builds on this by reorganising specialised units into larger systems, enabling coordination to operate at the scale of tissues and whole organisms rather than being confined to local domains.

### Integrated coordination

#### What is integrated coordination?

As mentioned above, most bilaterians and to a lesser extent cnidarians do not possess mere networks of neurons. In fact, nervous systems frequently come with varying degrees of integration from ganglia of various kinds found in both cnidarians and bilaterians, to the diverse array of nerve cords found across numerous bilaterian phyla. I am borrowing the notion of integration here directly from the conceptual apparatus of the TSC approach: integration is the process whereby existing units, typically ones that are already specialised, join together (on both ontogenetic and phylogenetic scales) to form new units at a higher level of organisation (e.g. cells to tissues or organs). Integrated coordination is distinguished from the previous stages by the assembly of specialised coordinators into higher-level structures, such as tissues and organs, enabling whole-organism control. The evolution of integrated nervous systems (as well as endocrine systems—see below) is readily explained in terms of the need for even greater efficiency for organism-wide coordination, especially in the context of the rapidly emerging complex food webs of the early Phanerozoic where animals needed to respond ever more quickly to the dangers of predation and the challenges of fast-escaping prey (Monk & Paulin, 2014; Roth & Dicke, 2013).

Integration of nervous system components can vary on a local/global spectrum. Local integration refers to integration at a local scale within the organism, thereby only connecting components within that part to each other; whereas global integration refers to integration at the scale of the whole organism, thereby connecting virtually all (or most) of the components of the organism (Madhani & Nejad Kourki, 2024; Nejad Kourki, 2022). In the present context, local integration is adaptively valuable if parts of the organism within which integration of specialised coordinators occurs need to coordinate their behaviour more or less independently of other parts of the organism; whereas global integration is adaptively valuable if all parts of the organism need to coordinate their behaviours together. Whether local or global, integration of specialised coordinators giving rise to higher levels of organisation (tissues and organs) enables much more rapid and more efficient processing of information due to the proximity of specialised coordinating units to each other and often the sensory and effector systems, which in turns allows for responding to the more complex challenges mentioned above (see example below).^11^

#### Integrated coordination in metazoan evolution

Across cnidarians and bilaterians, the various kinds of ganglionic aggregations exemplify local integration in the sense that they typically do not connect all parts of an organism and are instead localised to a part of the organism. The highly centralised nervous systems of vertebrates exemplify global integration because they do connect most parts of the organism, and the semi-centralised nervous systems of many bilaterians, with more than one nerve cord and often with ganglia alongside a central nerve cord, exemplify elements of both local and global integration. As mentioned above, most cnidarians (as well as members of several bilaterian phyla) exhibit a certain degree of integration in their nervous system in their circumoral nerve ring which coordinates the feeding and swallowing behaviour commonly found in cnidarians, which utilises a muscular pharynx surrounded by prey-catching tentacles. Many bilaterians, especially ones with one or more nerve cords running the length of their body, also exhibit some degree of integration of the nervous system around their mouth, which tends to be tightly associated with sensory organs such as tentacles or eyes on or around the head (Arendt et al., 2016; Martín-Durán et al., 2018). In many phyla this takes the form of a circumoral nerve ring which is likely homologous in some way to the corresponding cnidarian nerve ring, though the exact homology relationship is unclear. In other phyla, the ganglionic concentrations in the head have given rise to a highly recognisable organ: the brain, which is typically associated with an array of sense organs of several modalities (visual, chemical, electrical, etc.). In addition to this, some cnidarians also exhibit integrated sensory and nervous organs called rhopalia, which often contain eyes that have evolved independently from bilaterian eyes. All in all, though, bilaterians show on average much greater degrees of integration in their nervous systems than cnidarians, and the upper bound of integration is hardly comparable between the two groups. This both explains and is readily explained in terms of the much more active lifestyle of the bilaterians which, as mentioned above, probably also explains their success.

It is important here not to forget about the endocrine system. Under specialised coordination, I briefly discussed the endocrine system as another system for organism-level coordination besides the nervous system. Here, I wish to more clearly distinguish between the mere presence of endocrine cells and their integration into endocrine tissues and organs. Familiar examples include the various glands of vertebrates such as the pituitary gland or the pancreas, as well as the hepatopancreas of crustaceans (including insects^12^), as well as the highly ubiquitous sex glands found across most bilaterian phyla. The nervous system itself, or parts thereof, also typically play endocrine roles—just consider the hypothalamus/pituitary system in the vertebrates.^13^ Together, the nervous and endocrine system form the paradigmatic organ systems that perform coordinative roles in animals.

### Beyond integrated coordination

The MTEC framework, as briefly discussed above, identifies transitions beyond the mere centralisation of the nervous system. But whereas the first two transitions in this framework—namely the emergence of networks of neurons and centralisation of the nervous system—correspond closely to the emergence of specialised and integrated coordination, the two approaches diverge afterwards. Recall that the later transitions in the MTEC approach are the emergence of recurrence, lamination, and reflection in nervous systems (Barron et al., 2023): these at best loosely correspond to the emergence of structural complexity and higher levels of organisation, which is the focus of the TSC approach. What, if anything, might be regarded as further TSCs in nervous (and perhaps endocrine) system? In other words, *what lies beyond integrated coordination*?

The evolution of complex nervous systems in any animal clade is beyond my expertise and the scope of the present paper alike, and so I will only touch on this question briefly. In basic terms, nervous systems go beyond and build on ‘basic’ integration via the evolution of integrated units of organisation *within* the globally-integrated centralised nervous system, in turn via further rounds of modularisation, subfunctionalisation, and integration. Consider, for example, the familiar mammalian brain: though it might start out as the enlarged and partially folded anterior part of the dorsal nerve tube, it soon gives rise to distinct modules organised along the anterior–posterior axis, which in turn fold onto each other and each gives rise to further distinct parts.^14^ The final result is a brain that is organised into multiple levels and not just a diversity of cell types, but also a diversity of kinds of tissues and organ parts, reflected in readily distinguishable parts and subparts such as the cerebral cortex and its regions, the cerebellum, the midbrain, etc. While I am only using one example here, I hope that it goes to show the inherent promise of this approach for investigating the evolution of the nervous system across metazoan phylogeny.

## Conclusion

This paper has argued that applying the TSC approach to early metazoan evolution provides new conceptual traction on long-standing questions about the evolution of coordination and its relationship to cognition. By framing coordination as a transferrable function—not inherently tied to any specific structure or taxon—we are able to track how systems for adaptive regulation emerge and evolve across levels of biological organisation. The three stages described—collective, specialised, and integrated coordination—represent cumulative transitions in how coordination is implemented, from distributed consensus among undifferentiated units to tightly integrated systems of specialised processors such as neurons and endocrine cells.

This schema complements the MTEC approach in two key ways. First, it situates the evolution of nervous and endocrine systems within a more general account of functional reorganisation across levels of organisation, as captured by the TSC framework. Second, it adds conceptual depth to the MTEC narrative by introducing a ‘transition zero’: the emergence of coordination at the scale of the multicellular organism prior to the evolution of dedicated networks (i.e. collective coordination). This move also helps clarify the relationship between coordination and cognition, avoiding both overextension and unnecessary restriction of cognitive categories by grounding them in concrete structural transformations.

Taken together, these contributions support a more integrated view of cognitive evolution—one that links structural and functional complexity with emerging capacities for systemic control. They also illustrate how theoretical tools developed for studying transitions in biological organisation can shed light on the origins of cognition itself.

## Data Availability

Not applicable.

## Footnotes

1 I acknowledge that a proponent of plant cognition may disagree with this—after all, much of plant ‘behaviour’ is developmental in nature (e.g. phototropism, gravitropism, chemical defence, etc.). Nevertheless, the outlook of this paper is admittedly zoocentric, and highlighting the natural boundary between more reversible physiological processes and more stable developmental processes helps us understand the evolution of cognitive systems in animal evolution.

2 Deletion is less prominent than the other three processes in what follows, since it typically reshapes coordinative architectures by pruning or reducing existing modules rather than by generating new ones. For this reason, while it is important to acknowledge its role—for example, in the loss of the apical organ in some bilaterians—it does not feature as centrally in my analysis of the emergence of new forms of coordination.

3 This mode of feeding, construed broadly, is extremely common across the metazoan clade, and many animals rely on various mechanisms for controlling the flow of water for increasing their feeding efficiency. A nice example is that of rotifers feeding; see: https://www.youtube.com/watch?v=rEAlHx_edDU.

4 Though this hypothesis is supported by morphological evidence as well as numerous modern phylogenomic analyses, there is a running debate on whether an alternative (and mutually exclusive) hypothesis is correct—namely that ctenophores (comb jellies) are in fact the sister group to all other animals (Dunn et al., 2008; Laumer et al., 2018; Pisani et al., 2015; Zhao et al., 2019). For the purposes of this paper, I am choosing to mostly ignore this debate because ctenophores are a fast-evolving clade with a highly divergent set of traits, which means that whether they are the sister clade to all other metazoans or are situated somewhere within the eumetazoan clade their character polarities have little bearing on the overall scenario being presented here, despite their more general relevance to the evolution of neurons (Arendt 2021; Burkhardt et al., 2023; Moroz 2015; Moroz 2021; Whelan et al., 2017).

5 This is best demonstrated by experiments where homogenised sponge cells readily reaggregate to form a new sponge, reminiscent of the transient multicellulars that form by aggregation (e.g. cellular slime moulds); see: https://www.youtube.com/watch?v=N462jZFr13k.

6 Also see: https://www.youtube.com/watch?v=Xcbt3yjl25I.

7 Though they have long been regarded as a sister group to eumetazoans, more recent analyses have placed them as a sister group to cnidarians (Laumer et al., 2018).

8 While cnidarians lack true endocrine signalling, they do have what has been called endocrine-like signalling; see Tarrant (2005).

9 By ‘input–process–output’ I mean this in a neutral, biological sense—as a shorthand for signalling architectures involving detection, transformation, and response. This should not be read as an endorsement of the classical ‘sandwich model’ of cognition (Hurley 1998).

10 Both ectoderm and endoderm, though their concentration and subtypes can vary according to which epithelium they inhabit and the species (Arendt, 2021; Arendt et al., 2016).

11 In a similar vein, Bich, Pradeu, and Moreau discuss the organisation of the intercellular space as an important factor in multicellular organisms’ ability to carry out functions effectively (Bich et al., 2019).

12 Insects are, indeed, a subclade of the broader crustacean clade; see von Reumont et al. (2012), Legg et al. (2013), Sasaki et al. (2013), Eyun (2017).

13 Another notable example involves moulting in insects, where neurosecretory cells in the brain produce a hormone (prothoracicotropic hormone or PTTH) which stimulates the prothoracic glands to produce the hormone ecdysone, which in turn triggers the moulting process (Guo et al., 2008; Truman 2006).

14 What’s more, this is readily reflected in the ontogenetic trajectory of the mammalian brain; see Puelles et al. (2013)
